# Injuries in Female Futsal Players: A Systematic Review

**DOI:** 10.3390/sports12110311

**Published:** 2024-11-17

**Authors:** Luis Miguel Fernández-Galván, Carlos Hernández Santana, Carlos López-Nuevo, Jorge Sánchez-Infante

**Affiliations:** 1 Faculty of Physical Activity and Sport Science, European University, 28670 Madrid, Spain; luisdepucela@gmail.com (L.M.F.-G.); carlos.hersan01@gmail.com (C.H.S.); carlosenrique.lopez@universidadeuropea.es (C.L.-N.); 2 Faculty of Sport Science, Jaime I University, 12006 Castellon de la Plana, Spain; 3 Health and Sport Sciences University School (EUSES), Rovira i Virgili University, 43002 Amposta, Spain; 4 Faculty of Health Sciences, Universidad Francisco de Vitoria, 28223 Madrid, Spain; 5 Physiotherapy Research Group of Toledo (GIFTO), Faculty of Physiotherapy and Nursing, Universidad de Castilla-La Mancha, 45071 Toledo, Spain

**Keywords:** injuries, injury prevention, sports, five-a-side soccer, injury risk

## Abstract

Background: Injuries represent one of the most challenging scenarios for both athletes and teams. The aim of this systematic review was to examine the incidence and epidemiological data of injuries in female futsal players. Methods: A systematic search was conducted using PubMed, Scopus, SportDiscus, and Web of Science databases, and subsequently, nine studies were selected. Results: The most frequently damaged area is the ankle (28.15%), followed by the thigh (19.99%), knee (18.41%), and groin (17.26%), according to an analysis of nine studies, seven of which included data on professional futsal players and four of which included amateurs. Elite (28.62%) and amateur futsal players (27.06%) experience ankle injuries almost equally, whereas amateurs suffer thigh injuries (29.41%) far more often than elite athletes (13.71%). The most common injuries are strains (27.05%) and sprains (40.6%), with amateurs suffering from sprains more frequently (51%) than elite athletes (36.44%) and elite athletes suffering from strains more frequently (29.4%) than amateurs (20%). Conclusions: Ankle injuries are the most prevalent in female futsal players, with amateurs particularly prone to sprains. Based on these results, professionals in this field may identify injury patterns that could guide future prevention efforts specifically tailored to female futsal players.

## 1. Introduction

Female futsal is known for its fast pace and intense physical demands, with 60.6% of players experiencing injuries during a typical season [1]. Futsal is the official designation for the indoor variant of associated soccer, played with five players per side (one goalkeeper and four outfield players), and is recognized by the international governing body of soccer, the Federation Internationale de Football Association (FIFA) [2]. In Spain, female participation in futsal has increased but remains below 7% of total players [3]. Futsal games are held on a 40 × 20 m pitch and consist of two 20 min halves, with time stopped at each dead ball and unlimited substitutions [1,2]. During a futsal match, players encounter frequent collisions and face high-intensity physical demands, including sudden accelerations, decelerations, rapid directional changes, tackling, and kicking [4]. These physical actions heighten the risk of contact and collisions, while the combination of increased physical demands and performance-related psychological stress places futsal players at a significant risk of injury.

Recent studies have examined the external and internal load demands in female futsal, revealing how various factors influence player workload and performance. Using principal component analysis, key load variables are identified, with distinct patterns emerging for full matches compared to individual halves [5]. Typically, players experience decreased physical performance in the second half, evidenced by a reduction in total distance covered (from 1710 m to 1635 m) and in high-speed movements (from 15.6% to 14.7% of the total distance) [5]. Over the season, higher internal loads are observed during pre-season compared to in-season periods, with oscillatory patterns in later mesocycles [1]. At the internal load level, female futsal players exhibit high physiological demands, shown by elevated blood lactate concentrations (5.3 mmol∙L^−1^), near-maximal heart rate values (86.4%), and perceived exertion levels reaching 12 on the Borg scale (64–89% of maximum heart rate) [6]. Additionally, match analysis technology highlights the effects of fatigue on mechanical and locomotor activities. Research on youth futsal players also suggests that manipulating space and player numbers during training significantly influences both external and internal load demands, with smaller player numbers and larger relative areas generally increasing load [7].

Female futsal players face a significant risk of injury, with studies reporting injury rates of 54.1% to 87.73% among players [8,9]. The most common injuries occur in the lower extremities, particularly affecting the ankle, knee, and thigh [8,10]. Ankle sprains are the most prevalent injury, followed by ligament and meniscus lesions [9]. Non-contact injuries are more frequent than contact injuries, contrary to some previous findings [8]. Most injuries occur during training sessions rather than matches [8]. The injury burden is highest for knee injuries, followed by quadriceps and hamstring strains [10]. The injury risk peaks during the initial weeks of competition after pre-season and following the Christmas break [10]. To reduce injury prevalence, specific prevention strategies focusing on the ankle, knee, and thigh are recommended [8]. The review by Ruiz-Pérez et al. [2] analyzed injuries in futsal players, covering both male and female athletes, though with notable limitations. Firstly, the study included only six studies, some with small sample sizes, which impacted the consistency of injury estimates. Additionally, variations in injury definitions and data collection methods across studies complicated the comparability of findings. A lack of specific data also prevented detailed sub-analyses on injury location, type, and severity.

Considering the above research gap, the aim of this systematic review was to examine the incidence and epidemiological data of injuries in elite and amateur female futsal players. This review aims to establish a dedicated injury prevention strategy for professional female futsal players and, if needed, an additional strategy for recreational players.

## 2. Materials and Methods

The present systematic review was conducted in accordance with the Preferred Reporting Items for Systematic Reviews and Meta-Analyses (PRISMA) guidelines [11]. The review is registered in the International Prospective Register of Systematic Reviews (PROSPERO) under the registration number CRD42024571096.

### 2.1. Data Sources and Bibliographic Search Strategies

**Figure 1:**
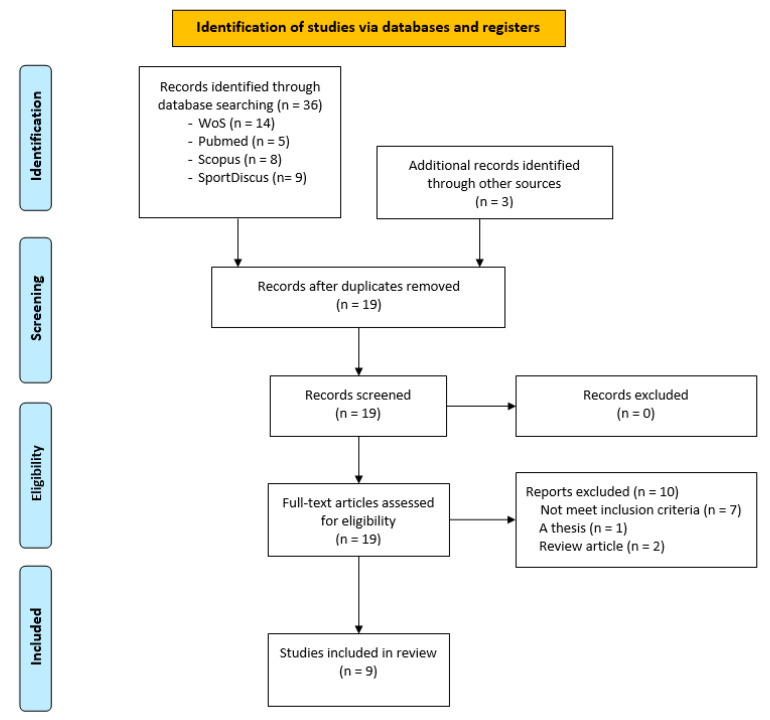
PRISMA flow diagram for the study search and selection process.

Four electronic databases, Web of Science (WoS), PubMed, Scopus, and SportDiscus, were searched from their inceptions until 31 March 2024. Articles were searched using Boolean combinations of the following keywords or equivalent Medical Subject Heading (MeSH) terms: (futsal [Title] or “indoor soccer” [Title]) and (female [Title] or wom * [Title]) and (injur * [Title]). Additionally, a manual search of the reference lists from published reviews was conducted to ensure that any potentially relevant studies missed during the electronic search were included. No language or other restrictions were applied to the initial search. Two reviewers independently assessed the relevance of studies by examining titles, abstracts, keywords, and full texts to determine their eligibility for inclusion in the systematic review (Figure 1). A standardized form was used to assess study eligibility, and in cases of disagreement, a third reviewer was consulted to resolve the topic. A full electronic search strategy for each database is available on request.

### 2.2. Study Selection

To be included in the present systematic review, studies had to have a strategy that satisfied the following inclusion criteria: (i) original articles that described the epidemiology of futsal injuries and (ii) studies conducted on female teams. Given the limited number of studies available, no strict exclusion criteria were applied initially. However, thesis papers, review articles, case reports, studies focused solely on specific injuries or biomechanics, and articles that did not meet all the inclusion criteria were excluded to maintain methodological rigor. Additionally, a manual search of references from selected articles was conducted to identify further relevant studies.

### 2.3. Data Extraction and Quality Assessment

Data extraction was conducted by two independent reviewers, with a third reviewer involved to resolve any disagreements when necessary. The data were organized into a standardized extraction form, capturing details such as the study design, participant characteristics, age, level of competition (elite or amateur), most commonly affected injury areas, and types of injuries reported. To assess the methodological quality of the included studies, the Strengthening the Reporting of Observational Studies in Epidemiology (STROBE) guidelines were utilized. The STROBE checklist, which consists of twenty-two items aimed at promoting clarity and transparency in reporting observational research, was applied to facilitate critical evaluation and reproducibility of findings. This checklist consists of twenty-two questions divided into six sections. The first section includes one item for the title and abstract; the second includes items two and three for the introduction; the third, ranging from items four to twelve, addresses the study methods; the fourth section on results includes items thirteen to seventeen; items eighteen to twenty-one examine the discussion; and item twenty-two addresses other information (funding). This quality assessment was conducted independently by two authors, with input from a third reviewer in cases of disagreement. In addition, the Kappa index was used to quantify the level of agreement between the two evaluators who each classified the items into categories.

## 3. Results

### 3.1. Study Selection

A flow diagram illustrating the article selection process is shown in Figure 1. Initially, a total of thirty-six articles were identified through database searches, along with an additional three articles from other sources. After removing duplicates (n = 20), 19 unique records remained. All 19 titles and abstracts were screened, and 19 full-text articles were assessed for eligibility. Of these, ten manuscripts did not meet the eligibility criteria, including seven that failed to meet inclusion criteria, one thesis, and two review articles. Ultimately, nine articles were included in this systematic review.

### 3.2. Methodological Quality

To evaluate the quality of the articles included in this review, the STROBE statement was used. In Supplementary Material Table S1, the Table of methodological quality of the studies analyzed using the STROBE tool can be found. All articles included in this study satisfied at least 18 and at most all of the 22 items that should be included in the reports of observational studies (STROBE statement).

Cohen’s kappa index was calculated to determine reliability between the authors involved in this process. The correlation found between the evaluators was exceptionally high, with a Cohen’s kappa index of 0.934. This result indicates an almost perfect agreement between the authors, suggesting that the evaluation process was highly reliable and consistent.

### 3.3. Participants, Age, and Level

The participants in the studies examined were from five different countries (Spain, Brazil, Portugal, Iran, and Italy), with ages ranging between 16 and 35 years old. Seven of the articles analyzed related to elite futsal players, and four contained information about non-elite (amateur) players (Table 1, Appendix A).

### 3.4. Most Common Injury Area

These percentages were calculated as arithmetic means of values reported in studies that included data for each specific injury area. The most common injury area was the ankle (28.15%), which was generally the most reported area, followed by the knee (18.41%), groin (17.26%), and thigh (19.99%). Taking into account the areas affected, we do not see significant differences. Among elite athletes, ankle injuries comprised 28.62% of the total, while they represented 27.06% among amateurs. Following this pattern, knee injuries comprised 18.91% of the total injury prevalence in elite futsal players versus 16.63% in amateur futsal players. On the other hand, groin injuries comprised a total of 17.26% in elite athletes, and no groin injury data were collected for amateur futsal players. Lastly, we do see a significant difference in thigh injuries (concerning hamstrings and quadriceps): for elite futsal players, the percentage was 13.71%, while in amateur players, it was 29.41%.

### 3.5. Most Common Injury Types

These percentages were calculated as arithmetic means of values reported in studies that included data for each specific injury types. Regarding the most common injury type, we highlight sprains and strains (sprains are injuries to muscles or tendons and strains are injuries to the ligaments and joint capsules), which ranged between 40.6% and 27.05%, respectively, of the total injuries. Following a comparison of elite and amateur futsal players, we observe an important variance. In elite futsal players, sprains comprised 36.44% of the total injuries, while in amateur players, this number grows to 51%. Strains also differ; in elite indoor soccer players, the incidence of strains was 29.4%, while in amateur players, it was 20%. Three of the nine studies did not collect data on the prevalence of injury types [8,9,10].

### 3.6. Most Common Injury Severity

The severity of injuries in futsal demonstrates considerable variability across studies, reflecting differences in classification methods and recovery timeframes. Serrano et al. [12] reported that minor injuries requiring 0–3 days of recovery comprised 9.1%, with slightly higher rates (12.7%) for injuries requiring 4–7 days. In contrast, Angoorani et al. [14] observed a higher proportion of injuries with short recovery periods, where 39.2% required 1–3 days, and 32.1% required 4–7 days. Prolonged injuries, such as those exceeding 28 days, varied significantly between studies, representing 10.7% of injuries [14], compared to 33.7% [8]. Additionally, injuries requiring 2–3 weeks were reported in 25.8% of cases [15], while injuries lasting 1–2 months accounted for 18.3%.

Regarding severity classifications, Ruiz-Pérez et al. [10] categorized injury severity based on incidence rates per 1000 h of exposure: minimal injuries (1.8/1000 h), mild injuries (1.9/1000 h), moderate injuries (2.7/1000 h), and severe injuries (0.22/1000 h). Similarly, Lago-Fuentes et al. [1] found that slight injuries represented 14.4% of cases; minor injuries, 27.7%; moderate injuries, 41.1%; and major injuries, 16.6%. Lago-Fuentes et al. [16] reported similar proportions, with minor injuries accounting for 29.8%; moderate injuries, 36.6%; and major injuries, 16.7%. Notably, some studies did not provide data on injury severity, emphasizing the need for standardized reporting in this area [9,13].

### 3.7. Most Common Injury Incidence

The incidence of injuries in futsal varies significantly across studies, reflecting differences in methodologies and reporting. Angoorani et al. [14] reported that an injury incidence of 4.17 injuries per 1000 h of exposure documented slightly higher rates of 5.0 injuries per 1000 h and 5.68 injuries per 1000 h [1,16]. Conversely, Ruiz-Pérez et al. [10] reported the highest incidence rate, at 6.74 injuries per 1000 h of exposure, indicating variability across studies in terms of injury occurrence. 

In studies where injury incidence was calculated per player, Uluöz [15] reported an average rate of 1.4 injuries per player. Notably, some studies did not provide specific data on injury incidence, which underscores the need for consistent and standardized data collection methods in future research [8,9,12,13].

## 4. Discussion

The main findings of this systematic review indicate that lower-limb injuries, particularly to the ankle and knee, are the most prevalent among female futsal players, consistent with previous studies in this population [1,12]. This injury pattern may be attributed to the specific demands of futsal, where the lower extremities are subject to continuous stress due to the need for ball control, rapid movements, and constant interaction with opponents [5].

The reduced size of the futsal court (40 × 20 m) and the possibility of unlimited substitutions throughout the game promote high-intensity physical actions [1,4]. This environment makes futsal one of the most physically demanding team sports, surpassing soccer, basketball, and handball in terms of physical demands [17]. Players maintain an average heart rate near 90% of their maximum, with a work-to-rest ratio of 1:1, frequent changes in locomotor activities every 3.3 s, and short recovery intervals of 20–30 s between high-intensity bouts [17]. As a result, futsal shows a high injury rate, with averages exceeding 20 injuries per 1000 h of play [18].

This high-intensity style of play, characterized by frequent collisions and substantial physical demand, increases stress on joints, particularly the ankle and knee, thus raising the risk of injuries to these areas [1,8]. Specifically, the ankle is at greater risk during actions such as dribbling and shooting [19], while the knee, located at the center of the lower limb’s lever arm, is particularly vulnerable during movements requiring abrupt changes in speed and direction [19].

Our results indicate that elite players presented an ankle injury incidence of 28.62% and a knee injury incidence of 18.91%, with similar rates observed among amateur players (27.06% and 16.63%, respectively). However, notable differences were found in injury types: sprains were significantly more common among amateur players (51%) compared to elite players (36.44%).

In comparison with male futsal players, both the affected body regions and the types of injuries are, in general, comparable between sexes [2,20]. Studies on elite male futsal players report a high prevalence of muscle strains (32%) and ligament sprains (29%), mainly affecting the groin (19%), thigh (17%), knee (19%), and ankle (15%) [2,20].

This similarity in the distribution and types of injuries suggests that futsal’s physical demands place a high level of risk on the same anatomical regions, regardless of gender [2,20]. However, the observed differences in sprain frequency between amateur and elite female players, compared with males, may reflect variations in physical preparation and game intensity, representing an area of interest for future research [20].

In our study, injury severity data showed a predominant occurrence of moderate injuries, requiring 8 to 28 days for recovery, accounting for 59.4% of cases, though this proportion varies in other studies, where moderate injuries represent between 36.6% and 41.1%. Severe injuries, necessitating more than 28 days for recovery, were less frequent in our sample, ranging from 10.7% to 33.7%, with an incidence of 0.22 injuries per 1000 h of play. This aligns with the common trend noted in the literature, where injury frequency generally decreases as severity increases [19,21]. Conversely, mild injuries (1–7 days) in our study were substantial, representing between 4.8% and 39.2% of cases, although criteria inconsistencies across studies may contribute to this variability [22].

Our results show an injury incidence in futsal players ranging from 4.17 to 6.74 injuries per 1000 h of play, closely aligning with the rates reported in previous studies that highlight futsal as a high-risk sport. This range is comparable to findings by Emery and Meeuwisse [23] who reported 4.45 injuries per 1000 h for futsal, and by Willick et al. [24], with 22.4 injuries per 1000 h. Though Willick’s [24] rate is notably higher, it may reflect differences in injury definitions and inclusion of training sessions as part of the practice time, which can significantly influence injury ratios across studies. Additionally, our observed rates fall within the common range for futsal (4.45 to 292.4 injuries per 1000 h) noted in the literature, with Junge and Dvorak [21] supporting the use of injury-to-practice-time ratios for the most accurate inter-sport comparisons.

Understanding the epidemiology and mechanisms of injury is essential for implementing effective prevention strategies in women’s futsal. Given that this systematic review indicates a high incidence of ankle and knee injuries specifically, sprains among amateur players and muscle strains among elite players, it is crucial to develop prevention programs targeting these vulnerable areas. For instance, Valle et al. [25] demonstrated the potential of a preventive program based on plyometric training and motor control exercises to improve ankle stability in semiprofessional female futsal players, emphasizing the importance of targeted interventions for this injury-prone area. A focus on strengthening and conditioning lower-limb muscles, particularly around the ankle and knee, could be effective in reducing the risk of sprains and muscle strains [8]. Additionally, implementing proprioceptive and joint stability programs in pre-season training could contribute to reducing ankle sprains, especially in players with a history of this injury [26]. Lago-Fuentes et al. [27] supported this approach, identifying the use of proprioceptive training and functional movement assessments as key injury prevention strategies commonly applied and valued by technical staff in professional futsal leagues. A controlled progression in training load, combined with adequate recovery times, is also advisable to prevent overuse injuries [28]. These elements, along with a structured warm-up prior to training and matches, will optimally prepare the musculoskeletal system for futsal’s specific physical demands [29]. Oliveira et al. [30] further highlighted the benefits of structured warm-up routines, which improve balance, eccentric strength, and joint stability while reducing the incidence of acute and lower-limb injuries in futsal players. Multicomponent strategies, combining core stability, flexibility, and proprioceptive training, have also shown promise for reducing injury risk in this high-demand sport [30].

This review has a clear limitation related to the quality of some of the articles studied. Although screening was conducted, some articles had no data concerning the injury type, or they generalized the injury area. Additionally, there was notable variability in study designs and injury definitions across the included studies, which affects the generalizability and comparability of our results. Differences in sample sizes, injury definitions, data collection methods, and participant levels (elite vs. amateur) likely influenced the reported injury types and frequencies. This heterogeneity limited our ability to conduct a meta-analysis, leading us to opt for a qualitative synthesis to identify general patterns. Future studies should consider adopting standardized methodologies and consistent definitions to enhance comparability and enable robust quantitative analyses, thereby providing a stronger evidence base for injury epidemiology in female futsal players.

## 5. Conclusions

Female futsal players, both elite and amateur, are exposed to a high risk of injury. In both cases, we see that the most reported area encompasses the lower extremities, with the ankle being the area with the highest risk of sustaining an injury, followed by the knee. On the other hand, regarding the type of injury, sprains seem to be the most common. We do observe some differences for contusions/direct-contact injuries. Based on these results, professionals in this field may identify injury patterns that could guide future prevention efforts specifically tailored to female futsal players.

## Figures and Tables

**Table 1:** Study characteristics.

Study	Type of Study	Participants Age	Tier	Most Common Injury Area	Most Common Injury Type	Injury Severity	Injury Incidence
Serrano et al. (2013) [12]	Observational Retrospective	127 players: (9 NT, 101 DT, 17 NL)	Elite and NL	Knee (49.2%) Hand (17.5%)	Sprains (48%) Ruptures (14.7%)	0–3 days: 9.1% 4–7 days: 12.7% 8–28 days: 59.4% >28 days: 18.8%	No data
Souza Filho et al. (2019) [13]	Observational, descriptive and cross-sectional	89 players in Brazilian first div.	Elite and NL	Ankle (20.51%) Groin (20.51%) Knee (17.94%)	No data	No data	No data
Angoorani et al. (2014) [14]	Prospective cohort	17 players on Iranian NT 20–31 years old	Elite	Ankle (42.8%) Groin (14.2%) Knee (17.8%)	Ligament R. (7.4%) Sprains (59.2%) Meniscus (7.4%) Strain (7.4%)	1–3 days: 39.2% 4–7 days: 32.1% 8–28 days: 17.8% >28 days: 10.7%	4.17 per 1000 h
Uluöz (2016) [15]	Observational study Retrospective	66 players 17–28 years old	Trained	Ankle (26.9%) Knee (21.5%) L. back. (16.1%)	No data	1–3 days: 10.8% 4–7 days: 34.4% 2–3 weeks: 25.8% 1–2 months: 18.3% >3 months: 10.8%	1.4 per player
Ruiz-Pérez et al. (2019) [10]	Prospective	39 players 24.1 ± 3.9 years old	NL	Ankle (13.3%) Hamstring (26.7%) Quad. (23.3%) Thigh (50%)	Muscle I. (66.7%) Ligament I. (20%) Tendon I. (6.7%)	Minimal: 1.8/1000 h Mild: 1.8/1000 h Moderate: 2.7/1000 h Severe: 0.22/1000 h	6.74 per 1000 h
Gayardo et al. (2012) [8]	Quantitative, descriptive with transversal outlining	135 players in Brazilian first div. 16–35 years old	Elite and NL	Ankle(28.9%) Knee (23.1%) Thigh (24%)	No data	0–6 days: 4.8% 7–28 days: 52.9% >28 days: 33.7% No reply: 8.6%	No data
Lago-Fuentes et al. (2020) [1]	Prospective cohort	89 players in Spanish 1st and second div.	Elite and NL	Ankle (20%) Quad. (22.2%) Knee (15.6%)	Contusions (13.3%) Sprains (34.5%) Strains. (40%)	Slight: 14.4% Minor: 27.7% Moderate: 41.1% Major: 16.6%	5 per 1000 h
Nemcic et al. (2016) [9]		95 players Serie A: 26.14 ± 5.9 Serie C–D: 25.61 ± 4.63 Amateur: 28.73 ± 6.24	Serie A: Tiers 3 and 4 Serie C–D: Tiers 2 and 3 Amateur: Tier 2	Serie A: Ankle (26.59%) Knee (23.4%) Thigh (7.45%) Serie C–D: Ankle (41.93%) Knee (19.35%) Thigh (9.68%) Amateur: Ankle (41.17%) Knee (11.76%) Thigh (8.82%)	Serie A: Fracture (14.81%) Lig/Ten (13.83%) Sprain (25.53%) Serie C–D: Fracture (16.13%) Lig/ten. (12.9%) Sprain (35.48%) Amateur: Cont. (20.59%) Muscle I. (14.7%) Sprain (35.29%)	No data	No data
Lago-Fuentes et al. (2021) [16]	Prospective cohort	14 teams of Spanish first and second div. 179 players. 2017–2019	Tiers 3 and 4	Ankle (20.4%) Quad. (22%) Knee (16.8%)	Ligament R. (4.7%) Sprain (27.7%) Strain (40.8%)	Slight: 16.7% Minor: 29.8% Moderate: 36.6% Major: 16.7%	5.68 per 1000 h

NT = national teams; DT = district teams; NL = national leagues; Div = division; L. back = lower back; Quad = quadriceps; R = rupture; I = injury; Lig/ten = ligament or tendon; h = hours.

## Data Availability

Not applicable.

## Supplementary Materials

The following supporting information can be downloaded at https://www.mdpi.com/article/10.3390/sports12110311/s1: Table S1.

## Appendix A

This appendix includes Supplementary Materials relevant to the methodological rigor of the review but not essential to the flow of the main text. For instance, Table 1 provides an assessment of the methodological quality of the studies included in the review, analyzed using the STROBE tool. Although integral to understanding the robustness of the studies analyzed, this Table has been moved to the Supplementary Materials to streamline the main manuscript. The Table can be accessed in Supplementary Material Table S1 for further reference.
