# Supplementary material for: Injuries in Female Futsal Players: A Systematic Review
Source: Sports (Basel). 2024 Nov 17;12(11):311. doi: 10.3390/sports12110311 (PMC11598481; doi:10.3390/sports12110311)
Supplement: Supplementary file 1 [file sports-12-00311-s001.zip › sports-3296367-supplementary.pdf]

	Serrano et al., (2013) [12]	Souza Filho et al., (2019) [13]	Angoorani et al., (2014) [14]	Uluöz (2016) [15]	Ruiz-Pérez et al., (2019) [10]	Gayardo et al., (2012) [3]	La-go-Fuentes et al., (2020) [1]	Nemcic et al., (2016) [9]	Lago-Fuentes et al., (2021) [16]
1	√	√	√	√	√	√	√	√	√
2	√	√	√	√	√	√	√	√	√
3	√	√	√	√	√	√	√	√	√
4	√	√	√	√	√	√	√	√	√
5	√	√	√	√	√	√	√	√	√
6	√	√	√	√	√	√	√	√	√
7	√	√	√	√	√	√	√	√	√
8	√	√	√	√	√	√	√	√	√
9	√	X	√	√	X	√	X	X	√
10	√	√	√	√	X	√	√	X	√
11	√	√	√	√	√	√	√	√	√
12	√	√	√	√	√	√	√	√	√
13	√	√	√	√	√	√	√	√	√
14	√	X	√	√	√	√	√	√	√
15	√	√	√	√	√	√	√	√	√
16	√	√	√	√	√	√	√	√	√
17	√	√	√	√	√	√	√	√	√
18	√	√	√	√	√	√	√	√	√
19	√	√	√	X	√	X	√	X	√
20	√	√	√	√	√	√	√	√	√
21	X	√	√	X	√	√	√	√	√
22	X	X	√	X	√	X	√	X	√
Total	20	19	22	19	20	20	21	18	22
